# Determination of Pigments in Virgin and Extra-Virgin Olive Oils: A Comparison between Two Near UV-Vis Spectroscopic Techniques

**DOI:** 10.3390/foods8010018

**Published:** 2019-01-07

**Authors:** Eleonora Borello, Valentina Domenici

**Affiliations:** Dipartimento di Chimica e Chimica Industriale, via Moruzzi 13, 56124 Pisa, Italy; bore.89.ele@gmail.com

**Keywords:** extra-virgin olive oil, EVOO, chlorophylls, carotenoids, pigments, colour, quality, spectroscopy, ultraviolet-visible light, light absorption

## Abstract

The colour of olive oil is due to the presence of natural pigments belonging to the class of carotenoids, chlorophylls, and their derivatives. These substances, other than being responsible for the colour, an important qualitative feature of the oil, have antioxidant and, more generally, nutraceutical properties and their quantification can be related to the product’s quality and authenticity. In this work, we have quantified the total amount of carotenoids and chlorophylls’ derivatives in several virgin and extra-virgin olive oils produced in Italy, by using two different methods that are based on near-ultraviolet-visible absorption spectroscopy. The first method defines two indexes, K670 and K470, related to absorbance values of oil at wavelengths of 670 and 470 nm, respectively. The second method is based on the mathematical deconvolution of the whole absorption spectrum of the oil to obtain the concentrations of four main pigments present in olive oils: β-carotene, lutein, pheophytin A, and pheophytin B. The concentrations of the total carotenoids and total chlorophylls’ derivatives, as obtained by the two spectroscopic methods, are compared and the results are discussed in view of the practical usefulness of spectroscopic techniques for a fast determination of pigments in olive oil.

## 1. Introduction

Virgin olive oil is obtained exclusively from the fruits of the olive tree, *Olea Europaea* L., by mechanical extraction processes at controlled thermal conditions which do not lead to chemical and physical deterioration of the oil, thus preserving its characteristic and distinctive properties [1]. Virgin olive oil is an edible oil greatly appreciated, which is an essential component of the Mediterranean diet. Due to the high content of mono-unsaturated fatty acids, such as the oleic acid, and bioactive minor compounds, extra-virgin olive oil is considered beneficial for human health [2,3,4]. International recognized institutions and approved regulations, such as the International Olive Council (IOC), *Codex Alimentarius*, and the European Commission, have established a commercial classification based on both chemical-physical and organoleptic properties of the final product. Virgin olive oils (VOOs) and extra-virgin olive oils (EVOOs) are those ones having the highest content of minor compounds with bioactive and nutritional properties (about 1%–2% of the total weight of olive oil) [3,4]. This class of chemical compounds can be divided into polar phenols and their derivatives, and non-polar compounds, such as squalene and other triterpenes, sterols, tocopherols, and pigments [1,2,4,5,6,7,8].

Pigments determine olive oil’s distinctive colour. Their relative chemical composition varies during olive oil’s life and it depends on many factors, such as the type of cultivar (genetic factor), the climatic and environmental conditions, the state of ripeness of the fruit at harvest, the storage and sampling of olives, the oil production process, and the storage conditions of the final product [9]. Pigments, which are exclusively synthesized from plants and assimilated by humans only through the diet [10], can be divided in two main classes: Carotenoids and chlorophyll derivatives [9,11,12,13]. Olive oils contain a relatively rich variety of carotenoids (i.e., β-carotene, lutein, violaxanthin, neoxanthin, and other xanthophylls in minor percentages) and chlorophyll derivatives (i.e., chlorophylls A and B, pheophytins A and B, and other minor derivatives) [9,11,12,13]. Several works have demonstrated the potential health benefits of both carotenoids and chlorophylls’ derivatives [1,2,10].

In the literature, there are many papers and reviews relating the concentration of main pigments and other derived quantities (i.e., the ratio between lutein and β-carotene, or the relative ratio between lutein and minor carotenoids), with olive oil authenticity and quality [9,11,12,13,14,15,16,17,18,19,20,21,22,23,24,25,26,27,28]. Moreover, several works demonstrated the usefulness of pigments’ determination to reveal olive oil adulterations [29,30,31]. The identification and quantification of single pigments is usually performed by means of chromatographic methods, such as high performance liquid chromatographic with ultraviolet-visible detection (HPLC-DAD) [12,14,15,22,23,25,26,27]. On the other hand, the near UV-vis spectroscopic absorption technique has been used mainly to evaluate the total amount of carotenoids and the total amount of chlorophylls’ derivatives from absorbance values obtained on olive oil samples diluted in cyclohexane, as first reported by Mínguez-Mosquera et al. [11,13]. Despite an initial treatment of the oil sample, implying a dilution in cyclohexane, this method is relatively simple, fast, and cheap. For these reasons, this simple spectroscopic method has been used in several works [11,13,32,33] to determine the total concentrations of carotenoids and chlorophylls in view of a chemical-physic characterization of olive oils and their quality.

A recent new spectroscopic method, based on the quantitative analysis of the whole absorption spectrum of olive oil samples in the near UV-vis range from 390 nm to 720 nm, has been developed to determine the concentration of four main pigments: β-carotene and lutein among the carotenoids, and pheophytin A and pheophytin B among chlorophylls’ derivatives [17,19]. The advantage of this spectroscopic method is the very fast analysis and the absence of any sample treatment: Spectra are indeed acquired in bulk and they can be analysed by using a simple deconvolution procedure [17,18]. This spectroscopic approach has been recently tested on extra-virgin olive oil samples produced in several Mediterranean countries, from different cultivars, and it was validated by comparing it with the standard HPLC-DAD method [18], confirming its validity, goodness, and high reproducibility. Moreover, it was revealed to be useful in studying the effect of the different harvest years on the main pigments’ content of several extra-virgin olive oils produced from a blend of three cultivars (*Moraiolo*, *Frantoio*, and *Leccino*) typical of Tuscany (Italy) [24].

Other methods based on the analysis of near UV-vis spectra of virgin and extra-virgin olive oils have been developed in the recent years, either in combination with multivariate chemometric approaches [16,28,34,35,36,37] or by using neural network to the spectral analysis [29,30,36]. These methods provided are very useful for olive oil quality and authentication purposes, but their application requires a relatively complex data treatment and/or specific software. On the other hand, the need for fast, non-destructive, and validated analytical methods is justified by the raising competition in the field of olive oil and the consumer demand of high standards and high quality products.

The present work focuses on the determination of the total amount of chlorophylls’ derivatives, the total amount of carotenoids, and the total amount of pigments in several virgin and extra-virgin olive oil samples, either mono-cultivar or a blend of different cultivars, produced in Tuscany (Italy), in different harvest years. Two relatively simple near UV-vis spectroscopic approaches have been used: The first method, proposed by Mínguez-Mosquera et al. [11], applied to olive oil samples diluted in cyclohexane, and the second method, proposed by Domenici et al. [17], for the quantification of the four main pigments, applied to not fresh olive oils analysed in bulk. Results obtained by the two spectroscopic methods are then compared and discussed in view of their practical usefulness for a fast determination of pigments in virgin and extra-virgin olive oils.

## 2. Materials and Methods

In the following subsection, the olive oil samples investigated in this work, the experimental procedures, the analytical methods, and the mathematical tools employed are described.

### 2.1. Samples

In this research, several samples of Italian olive oils were analysed. Some of them were certified as extra-virgin olive oils according to the European Regulation (Reg. CE 1234/2007, annex). Both mono-cultivar and a blend of different botanic varieties, as well as samples harvested in different years (2012, 2015), were considered, as described in the following part.

Mono-cultivar extra-virgin olive oils come from four varieties of olives typical of Tuscany: *Frantoio*, *Leccino*, *Moraiolo*, and *Pendolino*, harvested at the end of October/beginning of November of 2015. These samples were provided by private producer companies in the coast area of Tuscany (LI, Livorno) [38]; some of them were stored at room temperature (≤22 °C) and in the dark, while others were stored in a refrigerator at a temperature of 4 °C (see Table 1). Olive oils made from a blend of cultivars were produced in 2012 or in 2015; they were stored in the dark at temperatures indicated in Table 1. Labels of different EVOO and VOO samples are also shown in Table 1.

### 2.2. Methods

The UV-vis absorption spectra of the olive oil samples were acquired by using a UV-vis spectrophotometer (Jasco V-550), at room temperature, by using quartz cells having an optical path of 1 cm (first method) or 0.5 cm (second method). Both experimental spectra of the olive oil samples in bulk and diluted in cyclohexane were collected in the range between 220 and 800 nm. All measurements were performed with three replicates.

The first method, described for the first time by Mínguez-Mosquera et al. [11], provides the total amount of carotenoids and chlorophyll derivatives, expressed in ppm. This model is based on the calculation of two indexes, namely K670 and K470, related to absorbance values of the olive oil, diluted in cyclohexane, at a wavelength of 670 nm and 470 nm, respectively. According to this method, the index, K670, provides a quantitative evaluation of the total content of chlorophylls (and their derivatives), since the absorbance of the olive oils at 670 nm is due exclusively to the presence of this fraction of pigments. Since pheophytin A is the major component of this fraction in typical olive oils [1,2,3,9,10,11,12,13], the total content of chlorophylls is expressed in terms of this compound, by using its extinction coefficient, ε, at a wavelength of 670 nm, as determined in a diluted solution of the pigment in cyclohexane, namely ε = 613 [11]. On the other hand, according to this method, the index, K470, assesses the total content of carotenoids, since the absorbance of olive oils at 470 nm is largely determined by carotenoids. The total content of carotenoids is expressed in terms of lutein, since it is the main carotenoid pigment present in olive oils, and its extinction coefficient, ε, at 470 nm, in a solution of ethanol, is calculated from the literature [11], and it is ε = 2000. Considering the high value of the coefficients of the extinction of lutein, to respect the linearity of the Lambert-Beer law, the method [11] involved the olive oil sample dilution as follows: 7.5 g of olive oil is exactly weighted and dissolved in cyclohexane, bringing to a final total volume of 25 mL. Once the absorption spectrum was obtained, the chlorophylls’ total fraction (C_Ch_tot_) and carotenoids’ total fraction (C_Ca_tot_) are calculated from the absorbance values at 670 nm ($A_{670}$) and 470 nm ($A_{470}$), respectively, and expressed in ppm (mg of pigment in 1 Kg of oil), by using the following equations:

(1) $$C_{\mathrm{Ch}\_\mathrm{tot}}\;(\mathrm{Total}\;\mathrm{chlorophylls})=\frac{A_{670}\cdot 10^{6}}{613\cdot 100\cdot d}$$

(2) $$C_{\mathrm{Ca}\_\mathrm{tot}}\;(\mathrm{Total}\;\mathrm{carotenoids})=\frac{A_{470}\cdot 10^{6}}{2000\cdot 100\cdot d}$$

where *d* corresponds to optical path length of the cell (1 cm).

The second method, recently developed [17,19], allows us to determine the main pigments in olive oil by analysing the near UV-vis absorption spectra of olive oils recorded in the bulk, without any dilution or sample treatment. Only in few cases, before recording the spectra, the samples are centrifugated for 30 min at 5000 rpm, to minimize the light scattering phenomena due to eventual suspended particles and micro-emulsion scattering effect. This is particularly useful in the case of non filtered olive oils. Quartz cells with 0.5 cm optical path length are used. The experimental spectra are recorded and analysed by using a mathematical tool, compatible with Excel, developed by Domenici et al. [17]. This approach, described in detail and optimized in previous works [17,18,19,20,21,24], allows us to determine four main pigments: β-carotene, lutein, pheophytin A, and pheophytin B.

This mathematical approach consists in the deconvolution of the experimental spectrum in terms of four orthogonal functions obtained from the original experimental spectra of the four main pigments diluted in triolein [17,18]. The fitting procedure gives us the concentration of the four pigments and other relevant parameters, such as the ratio between the total amount of carotenoids and chlorophyll derivatives, the percentage of lutein with respect to the carotenoid fraction, and so on. The main approximation of this approach, as previously described [17,18,19,20,21,24], is that eventual additional pigments present in olive oils are neglected. This aspect is particularly critical in the case of fresh olive oils, where the amount of chlorophyll A is relatively high [39,40,41]. In the present study, all samples were not fresh and they were analysed far beyond olives’ pressing, thus excluding this problem.

**Figure 1:**
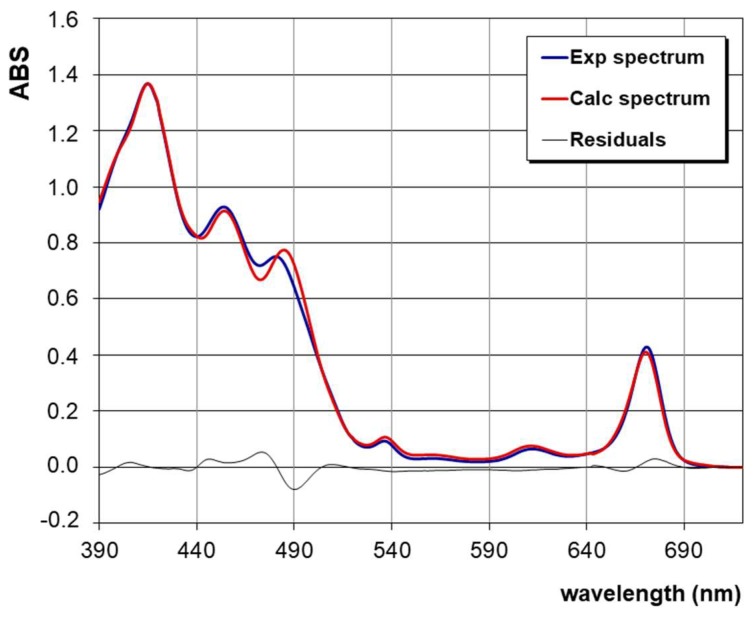
Example of near UV-vis absorption spectrum of an extra-virgin olive oil (EVOO) sample (namely T1), recorded in the range of 390–720 nm. Experimental (blue) and calculated (red) curves are reported with the residuals (black) curve.

As an example, the experimental spectrum of an EVOO sample (labelled T1) analysed in this work is reported in Figure 1 (blue curve) together with the calculated spectrum (red curve) from the deconvolution method. The residuals can also be visualized in Figure 1 as a black curve. The goodness of the mathematical treatment can be verified by the “R-square” test (R^2^), which estimates the correlation between the experimental values and the values predicted by the deconvolution procedure. In the case of the spectrum reported in Figure 1, R^2^ is 0.9978.

As previously reported [17,18,19,20,21,24], this method can be considered robust, with high reproducibility and good sensitivity in the case of not fresh olive oils (at least after three months from their production). For each olive oil sample, the near UV-vis spectra were measured with three replicates, and the values of concentration of the four pigments are expressed as the average value ± confidence intervals (over three replicates).

### 2.3. Statistical Analysis

The comparison between the two spectroscopic methods was performed concerning the concentrations of the total carotenoids and total chlorophylls’ derivatives as determined by the two approaches, by using the *t* test model within the EXCEL program. All parameters were determined in triplicate. The data reported were subjected to analysis of variance and were expressed as mean ± confidence interval (CI) of three measurements. Significant differences between values of all parameters were determined at *p* ≤ 0.05 according to the least significant difference (LSD) test.

## 3. Results and Discussion

In this work, olive oil samples were investigated in order to determine the main pigments’ content. All samples, EVOO and VOO ones, were analysed in different times after being stored at controlled temperatures and in the dark. In general, all investigated samples are relatively old, thus excluding the presence of chlorophylls. Instead, all chlorophylls can be considered fully converted in pheophytins (coloured) or other derivatives (not coloured). Since the object of the present work is the comparison between two spectroscopic (relatively fast and simple) methods, the samples were purposely selected in order to have both mono-cultivar and blend samples. Moreover, for the same reason, olive oil samples obtained in different harvesting years were selected.

First, the total content of chlorophylls’ derivatives and the total content of carotenoids were determined by applying the spectroscopic method proposed by Mínguez-Mosquera et al. [11], by using Equations (1) and (2), as described in the previous section. Different trials were performed to check the dilution effect (intra and inter-days tests), showing no significant changes. All measurements were performed in triplicate and the results are reported in Table 2. The sum of pigments (i.e., carotenoid and chlorophylls’ derivatives fractions) is also reported, showing a sensitive variability within the set of olive oil samples.

**Figure 2:**
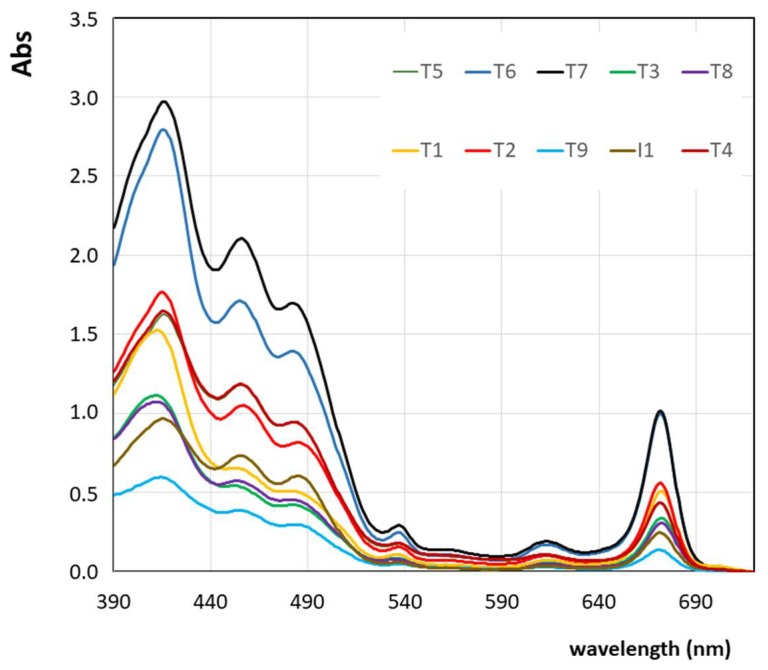
Superposition of experimental near UV-vis absorption spectra of the EVOO and VOO samples under investigations, as indicated in Table 1 and on the legend. The absorption spectra are scaled in order to have zero absorption (Abs = 0) at wavelengths larger than 720 nm.

All EVOO and VOO samples were analysed in the bulk by applying the second method (the one proposed by Domenici et al. [17]). Spectral measurements were performed for all samples the same day as the measurements described above by using the first method. For each sample, the amount of pigments is indeed supposed not to change within the same day. The near UV-vis absorption spectra recorded for all investigated samples in the bulk, in the range between 390 nm to 720 nm, as described in Section 2.2, are reported in Figure 2 (here, only one of the three replicates is shown, for each sample). All spectra were scaled in order to have zero absorption (Abs = 0) at wavelengths larger than 720 nm.

From Figure 2, it is evident the large variability of near UV-vis absorptions, due to different pigments’ content among the selected samples, which is an important issue for the purpose of the present work. The mathematical deconvolution of the absorption spectra recorded in the bulk and obtained by the fitting procedure proposed by Domenici et al. [17] gave us the concentrations of the four main pigments: Lutein, β-carotene, pheophytin A, and pheophytin B. The total amount of carotenoids is calculated as the sum between the lutein and β-carotene concentrations; while the total amount of chlorophylls’ derivatives is calculated as the sum between the pheophytin A and pheophytin B concentrations. The obtained values of the total amount of chlorophylls’ derivatives, total amount of carotenoids, and the sum of pigments are reported in Table 3. The goodness of the fitting procedure (and of the method proposed by Domenici et al. [17]) is expressed as the average value of R^2^ over a triplicate for each sample, and it is reported in Table 3.

From data reported in Table 2 and Table 3, it is evident there is large variability among samples, concerning both carotenoids’ and chlorophylls’ derivatives’ fractions. In particular, the olive oil samples richest in pigments (both carotenoids’ and chlorophylls’ derivatives) are the ones produced from the Moraiolo cultivar (sample T4) and blend samples mainly obtained from Moraiolo olives (samples T5-T7) and/or olive oil samples stored at low temperature (T = 4 °C) (samples T2, T5-T7). The samples, T6 and T7, have very high pigments’ content, reaching the total amount of 48.5 ppm (second method, Table 3) and 19.4 ppm (first method, Table 2). These samples have indeed both carotenoids’ and chlorophylls’ derivatives contents much larger than extra-virgin olive oil samples produced in the same geographic area, as reported in ref. [24]. On the other hand, the olive oil samples with the lowest content of pigments are two blend samples, I1 (EVOO) and T9 (VOO), with a sum of pigments, determined with the second method, of 14.1 ppm and 7.7 ppm, respectively.

**Figure 3:**
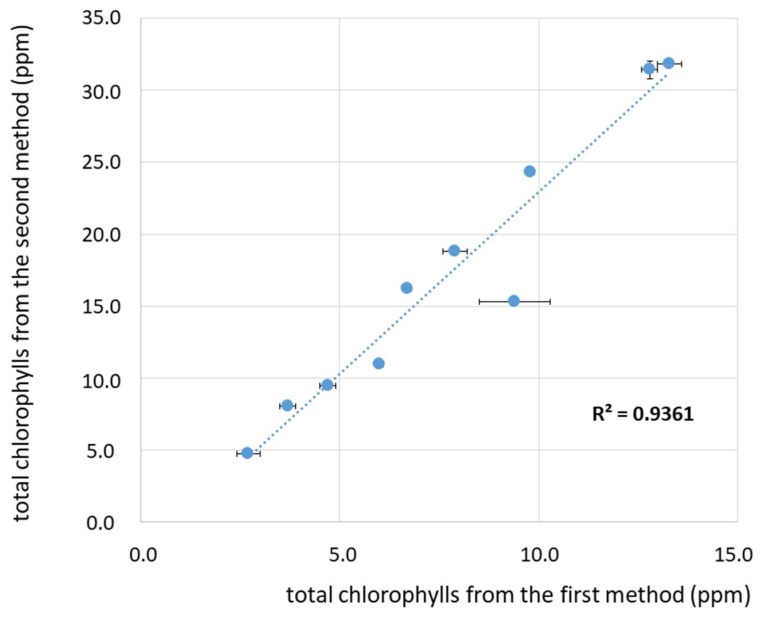
Plot of the amount of total chlorophylls (ppm) for the investigated olive oil samples as obtained from the two spectroscopic methods: Values obtained with the method proposed by Mínguez-Mosquera et al. [11] on the abscissa and by Domenici et al. [17] on the ordinate. A linear curve is shown, which correlates the two values, as described in the text. Each data point is displayed with an error bar corresponding to the confidence interval (CI). The R^2^ value corresponding to the linear regression fit is also reported.

**Figure 4:**
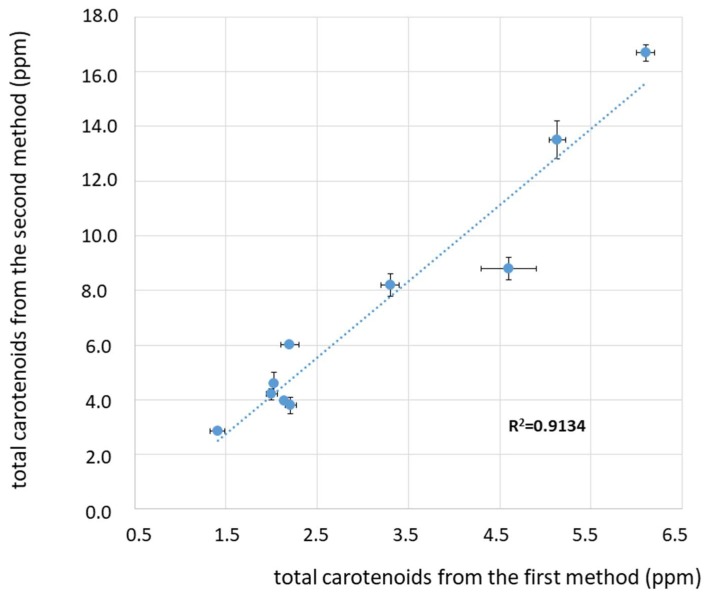
Plot of the amount of total carotenoids (ppm) for the investigated olive oil samples as obtained from the two spectroscopic methods: Values obtained with the method proposed by Mínguez-Mosquera et al. [11] on the abscissa and by Domenici et al. [17] on the ordinate. A linear curve is shown, which correlates the two values, as described in the text. Each data point is displayed with an error bar corresponding to the confidence interval (CI). The R^2^ value corresponding to the linear regression fit is also reported.

**Figure 5:**
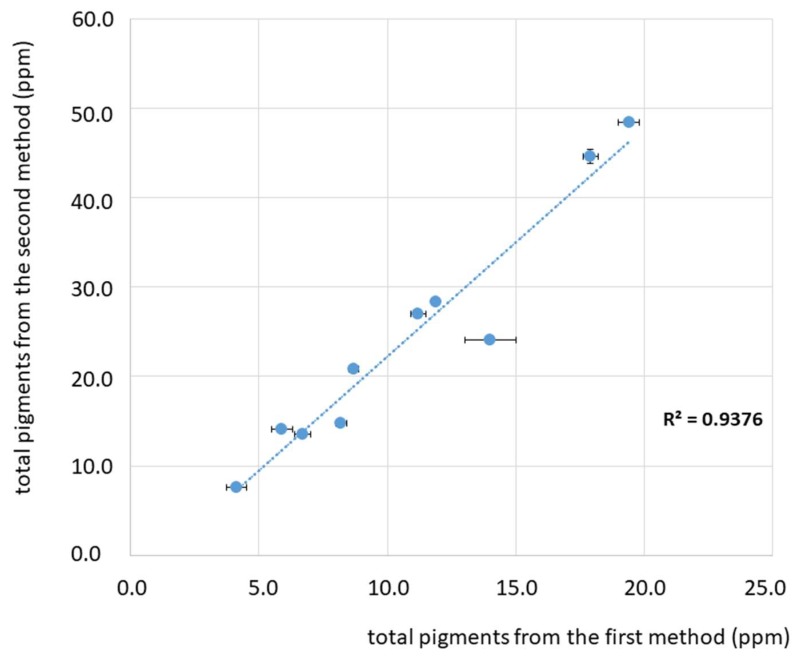
Plot of the sum of pigments (ppm) for the investigated olive oil samples as obtained from the two spectroscopic methods: Values obtained with the method proposed by Mínguez-Mosquera et al. [11] on the abscissa and by Domenici et al. [17] on the ordinate. A linear curve is shown, which correlates the two values, as described in the text. Each data point is displayed with an error bar corresponding to the confidence interval (CI). The R^2^ value corresponding to the linear regression fit is also reported.

To better visualize and discuss the pigments’ values obtained from the two spectroscopic methods, the total amount of chlorophylls’ derivatives, total amount of carotenoids, and the sum of pigments are reported in Figure 3, Figure 4 and Figure 5. The comparison between the values of total chlorophylls’ derivatives (Figure 3) obtained from the first and second methods demonstrate a good correlation, as shown by the linear regression (R^2^ = 0.9361). However, the method proposed by Mínguez-Mosquera et al. [11] gives values underestimated by about 40%–60% with respect to the second method proposed by Domenici et al. [17]. As shown in Figure 3, this behaviour can be generalized for the range of concentrations from about 5 ppm to about 32 ppm, which is a relatively high range considering the typical values of concentrations of chlorophylls’ derivatives in virgin and extra-virgin olive oils.

In Figure 4, the comparison between the values of total carotenoids obtained from the first and second methods is reported. In this case, data are much more scattered, and the error (indicated as confidence interval) associated to both methods is larger than for chlorophylls’ derivatives. As reported in a previous study, where the mathematical approach proposed by Domenici et al. [17] was analytically validated with respect to a standard HPLC-DAD protocol [18], the quantification of lutein is less straightforward due to the eventual presence of minor carotenoids having the same absorption spectrum than that of lutein, while β-carotene is quantified with very high precision [18]. This could be one reason for the worse correlation.

On the other hand, the quantification of the carotenoids’ fraction from the first method, from the sole absorbance value at 470 nm, seems not to be appropriate, since the contribution of the chlorophylls’ derivatives (in this case, mainly pheophytins A and B) to the spectral absorption at this wavelength is significant, as clearly demonstrated in refs. [17,18,19,20,21]. The correlation between the values of total carotenoids obtained by means of the two spectroscopic techniques is worse than for chlorophylls’ derivatives, as reported in Figure 4 (R^2^ = 0.9134). Moreover, the method proposed by Mínguez-Mosquera et al. [11] gives values underestimated by about 30%–60% with respect to the second method proposed by Domenici et al. [17].

The comparison between the sum of pigments obtained from the first and second methods is shown in Figure 5 and it demonstrates a relatively good linear correlation (R^2^ = 0.9376). However, as observed for the two pigments’ fractions, the method proposed by Mínguez-Mosquera et al. [11] provides values systematically underestimated by about 40%–60% with respect to the second method proposed by Domenici et al. [17].

In all cases, the differences between values obtained by the two spectroscopic methods are significant, according to the least significant difference (LSD) test (at *p* ≤ 0.05).

From these results, the application of Equations (1) and (2), as proposed by Mínguez-Mosquera et al. [11], seems not to be correct in order to get reliable values of the concentrations of the total carotenoids’ and chlorophylls’ derivatives in olive oils. In particular, the underestimation of both carotenoids’ and chlorophylls’ derivatives could be explained by observing the spectral contribution of the four main pigments to the near UV-vis absorption of olive oils [17,18,19,20,21]. In the region between 390 and 560 nm, both carotenoids’ and chlorophylls’ derivatives contribute to the spectrum, while the region between 630 and 700 nm can be safely assigned to the sole chlorophylls’ derivatives contribution. In a previous work, proposed by Cayuela et al. [16], the authors demonstrated that a more reliable determination of the total amount of carotenoids’ and chlorophylls’ derivatives, with respect to the simple calculation of the two K470 and K470 indexes, could be obtained by analysing a larger spectral region, from ultraviolet (UV) to near infra-red (NIR) wavelengths. However, their approach [16] implies the application of a more sophisticated multivariate model, as also proposed in other works, where the near UV-vis spectra are analysed to detect eventual adulterations of olive oils or to assess their authenticity and quality [29,30,36].

The calculation of the total amount of carotenoids and the total amount of chlorophylls’ derivatives from the main pigments’ content obtained by the method proposed by Domenici et al. [17] is more robust and reliable than the other spectroscopic method [11]. It implies the deconvolution of the spectrum of olive oil, which can be done simply by implementing a standard fitting program (by using, for instance, the molar extinction original data provided in ref. [18]. Moreover, this spectroscopic approach [17] has the advantage of avoiding any sample treatment or oil dilution, which represents a limitation if the analytical method is required to be fast and easy to be used by non-specialised operators. A disadvantage of this method, however, is related to the main approximation, which consists in neglecting the effect of eventual minor pigments (for instance, some minor carotenoids absorbing in the 390–520 nm region) and the not applicability of the method to fresh olive oils [39,40,41], where the presence of chlorophylls cannot be neglected.

Despite the significant difference between the two fast spectroscopic methods, the presence of a linear correlation suggests that the simple Equations (1) and (2) [11] could be corrected by introducing a numerical factor, but this aspect should imply the extension of this study to a much larger data set.

## 4. Conclusions

In this paper, several virgin and extra-virgin olive oils from Italy, mainly from the Italian central region of Tuscany, were studied to determine their pigments’ content, quantified by means of two different methods of analysis based on near-ultraviolet-visible absorption spectroscopy. Extra-virgin olive oil samples produced from cultivars typical of Tuscany, such as Frantoio, Moraiolo, Leccino, and Pendolino, were investigated in terms of pigments’ content. The first approach proposed by Mínguez-Mosquera et al. [11] defines two indexes, K670 and K470, related to single absorbance values at wavelengths of 670 and 470 nm, respectively. To our best knowledge, this method has never been validated, but, since it is relatively fast and simple, it has been widely used to estimate the total concentration of chlorophylls’ derivatives and the total concentration of carotenoids in several works on the chemical-physical characterization of olive oils [11,13,16,32,33]. The second approach, proposed by Domenici et al. [17], is based on a mathematical deconvolution of the whole near UV-vis absorption spectrum of the oil, recorded in the bulk without any sample treatment, to obtain the concentrations of four main pigments present in olive oils: β-carotene, lutein, pheophytin A, and pheophytin B. This spectroscopic method has been validated in previous works [17,18,19,20,21,24] and it can be considered a robust, precise, and reproducible spectroscopic analytical method for main pigments’ determination in not fresh olive oils. The main outputs of the present study, can be here summarized: 1. The calculation of the two K670 and K470 indexes according to the equations proposed by Mínguez-Mosquera et al. [11] underestimates both the content of carotenoids and the content of chlorophylls’ derivatives, with respect to the deconvolution method proposed by Domenici et al. [17]; 2. A good linear correlation between the values of concentration of chlorophylls’ derivatives obtained by means of the two methods was observed in a relatively large concentration range, which can be considered significant for virgin and extra-virgin olive oils; 3. In the case of the total amount of carotenoids, the linear correlation was worse, especially in the range between 1 and 3 ppm; 4. The statistical analysis of these data showed that the differences between the two methods were statistically significant; 5. The method proposed by Domenici et al. [17] is recommended for the quantification of the total amount of carotenoids and the total amount of chlorophylls’ derivatives in the case of not fresh olive oils, with respect to the method proposed by Mínguez-Mosquera et al. [11].

The optimization of new spectroscopic methods and their comparison in terms of pigments’ quantification, as reported in this work, are justified by the continued request of fast, cheap, and not-destructive methods to characterize olive oils and their quality. A further development is represented by the possibility to provide simple methods to be implemented in portable devices in order to check olive oils’ quality and authenticity at the service of consumers and producers.

## Figures and Tables

**Table 1:** Samples of Italian olive oils: Extra-virgin and virgin ones, mono-cultivar, and blend of different cultivars are indicated. The label of each sample, the geographic origin, and temperature of storage are also reported.

Label	Cultivar	Geographic Origin	Year of Harvesting	Storage Temperature	Classification ^1^
I1	blend	Italy ^2^	2015	≈22 °C	EVOO
T1	Frantoio	Italy, Tuscany (LI)	2015	≈22 °C	EVOO
T2	Frantoio	Italy, Tuscany (LI)	2015	≈4 °C	EVOO
T3	Leccino	Italy, Tuscany (LI)	2015	≈22 °C	EVOO
T4	Moraiolo	Italy, Tuscany (LI)	2015	≈22 °C	EVOO
T5	Blend ^3^	Italy, Tuscany (LI)	2012	≈4 °C	EVOO
T6	Blend ^3^	Italy, Tuscany (LI)	2015	≈4 °C	EVOO
T7	Blend ^3^	Italy, Tuscany (LI)	2015	≈4 °C	EVOO
T8	Pendolino	Italy, Tuscany (LI)	2015	≈22 °C	EVOO
T9	blend	Italy, Tuscany (FI)	2012	≈22 °C	VOO

^1^ The EVOO classification, where indicated, is based on sensory characteristics (International Regulations, Reg. CE 640/2008) and analytical indices (European Regulation, Reg. CE 1234/2007, annex). ^2^ This sample was a commercial one (Italian brand). ^3^ EVOO samples with a prevalence of Moraiolo cultivar.

**Table 2:** Values of total chlorophylls’ derivatives, total carotenoids, and sum of pigments, as determined by the first method (Mínguez-Mosquera et al. [11]). Values are reported as mean ± confidence interval (CI) of three measurements.

Label	Total Chlorophylls’ Derivatives (ppm)	Total Carotenoids (ppm)	Sum of Pigments (ppm)
I1	3.7 ± 0.2	2.2 ± 0.1	5.9 ± 0.2
T1	6.7 ± 0.1	2.03 ± 0.03	8.7 ± 0.1
T2	7.9 ± 0.1	3.3 ± 0.1	11.2 ± 0.1
T3	6.0 ± 0.1	2.2 ± 0.1	8.2 ± 0.1
T4	9.8 ± 0.9	2.14 ± 0.02	11.9 ± 0.9
T5	9.4 ± 0.3	4.6 ± 0.3	14.0 ± 0.3
T6	12.8 ± 0.2	5.1 ± 0.1	17.9 ± 0.2
T7	13.3 ± 0.3	6.1 ± 0.1	19.4 ± 0.3
T8	4.7 ± 0.2	2.0 ± 0.1	6.7 ± 0.2
T9	2.7 ± 0.3	2.4 ± 0.1	5.1 ± 0.3

**Table 3:** Values of total chlorophylls’ derivatives, total carotenoids, and sum of pigments, as determined by the second method (Domenici et al. [17]). Values are reported as mean ± confidence interval (CI) of three measurements.

Label	Total Chlorophylls’ Derivatives (ppm)	Total Carotenoids (ppm)	Sum of Pigments (ppm)	R^2^ (Fitting Method)
I1	8.1 ± 0.1	6.0 ± 0.1	14.1 ± 0.1	0.9836
T1	16.2 ± 0.2	4.6 ± 0.4	20.8 ± 0.2	0.9788
T2	18.8 ± 0.1	8.2 ± 0.4	27.0 ± 0.1	0.9846
T3	11.0 ± 0.1	3.8 ± 0.3	13.8 ± 0.1	0.9861
T4	24.3 ± 0.2	3.97 ± 0.04	28.3 ± 0.2	0.9875
T5	15.3 ± 0.1	8.8 ± 0.4	24.1 ± 0.4	0.9789
T6	31.4 ± 0.6	13.5 ± 0.7	44.5 ± 0.6	0.9782
T7	31.8 ± 0.1	16.7 ± 0.3	48.5 ± 0.3	0.9864
T8	9.5 ± 0.1	4.2 ± 0.2	14.7 ± 0.2	0.9852
T9	4.8 ± 0.1	2.9 ± 0.1	7.7 ± 0.1	0.9863
